# Object Segmentation and Ground Truth in 3D Embryonic Imaging

**DOI:** 10.1371/journal.pone.0150853

**Published:** 2016-06-22

**Authors:** Bhavna Rajasekaran, Koichiro Uriu, Guillaume Valentin, Jean-Yves Tinevez, Andrew C. Oates

**Affiliations:** 1 Max Planck Institute of Molecular Cell Biology and Genetics, Dresden, Germany; 2 Max Planck Institute for the Physics of Complex Systems, Dresden, Germany; 3 Theoretical Biology Laboratory, RIKEN, Saitama, Japan; 4 MRC-National Institute for Medical Research, London, United Kingdom; 5 Department of Cell and Developmental Biology, University College, London, United Kingdom; University of Campinas, BRAZIL

## Abstract

Many questions in developmental biology depend on measuring the position and movement of individual cells within developing embryos. Yet, tools that provide this data are often challenged by high cell density and their accuracy is difficult to measure. Here, we present a three-step procedure to address this problem. Step one is a novel segmentation algorithm based on image derivatives that, in combination with selective post-processing, reliably and automatically segments cell nuclei from images of densely packed tissue. Step two is a quantitative validation using synthetic images to ascertain the efficiency of the algorithm with respect to signal-to-noise ratio and object density. Finally, we propose an original method to generate reliable and experimentally faithful ground truth datasets: Sparse-dense dual-labeled embryo chimeras are used to unambiguously measure segmentation errors within experimental data. Together, the three steps outlined here establish a robust, iterative procedure to fine-tune image analysis algorithms and microscopy settings associated with embryonic 3D image data sets.

## Introduction

Cells within a developing multicellular organism undergo dramatic changes in gene expression, morphology and motion. Investigating these processes has been greatly enabled by recent advances in transgenesis to express a wide range of fluorescent reporters in the embryo [1]. It is now possible to visualize, to a certain degree, the spatio-temporal expression of protein levels at cellular and sub-cellular resolutions within developing embryos [2–6]. However, extracting and quantifying signals manually from large data sets is an exhausting and partially subjective task. Therefore, considerable efforts have been made over the last years to develop automated software for 3D segmentation of cellular objects.

The first hurdle in image analysis is to detect a distinct object within an image, a process referred to as segmentation. Segmentation assigns pixels either to the background or to an object. Separate objects may appear as a single fused object if the boundaries between them cannot be well resolved during imaging. This is particularly true in the *z*-dimension. Indeed, the point spread function of a typical objective extends far more in *z* than in *x* and *y*. In some embryonic contexts, the arrangement of cells in a 2D sheet greatly simplifies object segmentation, but much of the development occurs with a fundamentally 3D geometry. This is exacerbated by noise resulting from fast acquisition and low laser intensity, which is often the price to pay for decent temporal resolution and low photo-toxicity. Given the biological question and its embryonic context, one needs to test and strike a balance between spatial and temporal resolution during image acquisition. This needs to be supported by development of relevant, flexible, reliable, and automated image analysis tools. An image analysis protocol that addresses a specific biological question may even call for re-design of the image acquisition parameters to achieve better accuracy. However, to ensure confidence in such tools, it is essential that they be accompanied by objective measures of segmentation accuracy and straightforward protocols for optimizing image processing for specific problems [7–9].

One key to measuring accuracy of image processing is a ground truth data set: an image data set in which the true position of objects is known and, therefore, can be used to test the predictions of a segmentation algorithm [10]. Unfortunately, ground truth data sets do not exist for the majority of developmental imaging problems. Prior information, such as the expected number of nuclei, has been used to evaluate and constrain segmentation algorithms [6,11], but this approach is limited to early developmental stages or highly stereotypical processes.

Here, we describe an integrated imaging and image-processing protocol to segment the positions of cell nuclei in densely packed 3D embryonic tissues. Overlap of neighboring nuclear boundaries in an image is a major source of fused objects (under-segmentation). We developed an easily tunable object-separating algorithm with simple post-processing steps that was tuned and validated using synthetic data sets and chimeric dual-labeled embryos. The chimeric embryo assay allowed us to establish the *in vivo* “ground truth” data sets to assess the accuracy and reliability of object segmentation. We proposed the use of synthetic and *in vivo* ground truth data sets as a general strategy to optimize microscopy and evaluate algorithmic performance.

## Materials and Methods

### Ethics statement

All zebrafish (*Danio rerio*) husbandry and experimental procedures were performed in accordance with the German animal protection standards and were approved by the Saxonian Ministry of Environment under license number Az. 74–9165.40-9-2001 granted to the Max Planck Institute of Molecular Cell Biology and Genetics. Zebrafish were raised and kept under standard laboratory conditions. We used embryo medium (E3) with ethyl-m-aminobenzoate methanesulphonate (Tricaine) that prevents embryos from twitching during imaging. Embryos were sacrificed using 0.5% Tricaine to stop the heart.

### 4D image acquisition

We imaged the presomitic mesoderm (PSM) and tailbud of dechorionated *h2aflv-gfp*/ *h2aflv-mCherry* transgenic zebrafish embryos between 13 to 20-somite stages. Embryos were placed in agarose (1.5% low melting point-agarose in E3 without methylene blue and 0.02% Triciane) covered imaging dish with conical depressions such that the yolk fit laterally into the depression while the posterior body developed parallel to the agarose [12, 13]. An upright Zeiss confocal LSM with 40x/1.0 NA water dipping objective and 488 nm/561 nm laser light excitation (depending on the fluorophore) was used to obtain 2D images (8-bit/16-bit grayscale) of labeled nuclei parallel to the *xy* focal plane within the embryonic tissue. We typically imaged 512 × 512 pixels in *xy* and 20–30 overlapping z-slices, with voxel size of 0.691 × 0.691 × 1.75 μm^3^ respectively. A time series was recorded for 2 to 2.5 hours at a constant temperature ranging between 23°C and 28°C in the presence of 0.2 mg/mL Tricaine. Embryos were removed from the agarose after imaging and their normal development was verified until 30 hours under physiological conditions. An example image data is publicly available (S1 Dataset).

### Transplant experiments

Genetic mosaics were generated by cell transplantation as described previously [14, 15]. Donor embryos expressing two histone variants fused to GFP and mCherry (*h2AflV-gfp* / *h2AflV-mCherry*) were allowed to develop until blastula. Approximately 20 to 30 cells from the double-labeled donor embryos were transplanted into age-matched *h2AflV-mCherry* host embryos. The *h2AflV-mCherry* embryo chimeras were screened for *gfp* expression in the PSM and tailbud and were imaged using both 488 nm (GFP channel) and 561 nm (mCherry channel) lasers simultaneously as described in the previous section.

### Implementation of segmentation and synthetic image data

The algorithm was implemented in Matlab (R2011a to R2014b) using the Image Processing and Statistics toolbox, including built-in functions for K-means and GMM. All the results in the paper have been generated from the MATLAB codes, which is the reference implementation for this work (S1 Appendix). We also provide the community with a Fiji [16] plugin (http://sites.imagej.net/CWNS/) that recapitulates the MATLAB code, with the following differences: (1) nuclear splitting is solely based on K-means clustering operating on nuclear volume; (2) an optional tracking step is proposed [17]. The synthetic images were produced and analyzed using Mathematica (S2 Appendix). For details on segmentation algorithms and generation of synthetic images, see S1 Text.

### Measuring efficiency of segmentation algorithms using sensitivity and precision

#### Synthetic images

To measure error rates of the segmentation algorithms in synthetic images, each 3D centroid obtained by the algorithm was matched with each true position based on the Euclidian distances between them. We adopted a particle-matching algorithm and minimized the cost function defined as the summation of Euclidian distances between assigned pairs [18]. The segmentation algorithm was judged to have detected the object correctly if a match was found between the true position of an object and the segmented position within a radius of 2 μm. A miss was scored when a true position was not paired with any of the segmented centroid positions or an object was under-segmented. When a segmented centroid was not paired with any true positions, we considered the segmentation algorithm had detected false signals (e.g. noise) as an object or over-segmented an object. We counted the number of real objects correctly detected by the algorithm *N*_*ra*_ and defined sensitivity as *N*_*ra*_/*N*_*r*_ where *N*_*r*_ is the true number of objects in a synthetic image. We defined precision as *N*_*ra*_/*N*_*s*_ where *N*_*s*_ is the total number of segmented objects.

#### Chimeric Embryos

Segmentation of the sparsely distributed *h2AflV*-*gfp* positive nuclei gave error-free positions that were compared to the segmentation results of the densely packed nuclei. Because the centroid of a correctly segmented transplanted nucleus in the dense channel should correspond to a ground truth data point from the sparse channel, we checked for matching centroid position of nuclei based on Euclidian distance from sparse and dense channels within 2 μm (a typical nuclear radius was 4 μm). Sensitivity was then defined as the ratio of the number of matches between dense and sparse (true positive) and the total number of nuclei in the sparse channel (true positive + false negative).

## Results

**Fig 1:**
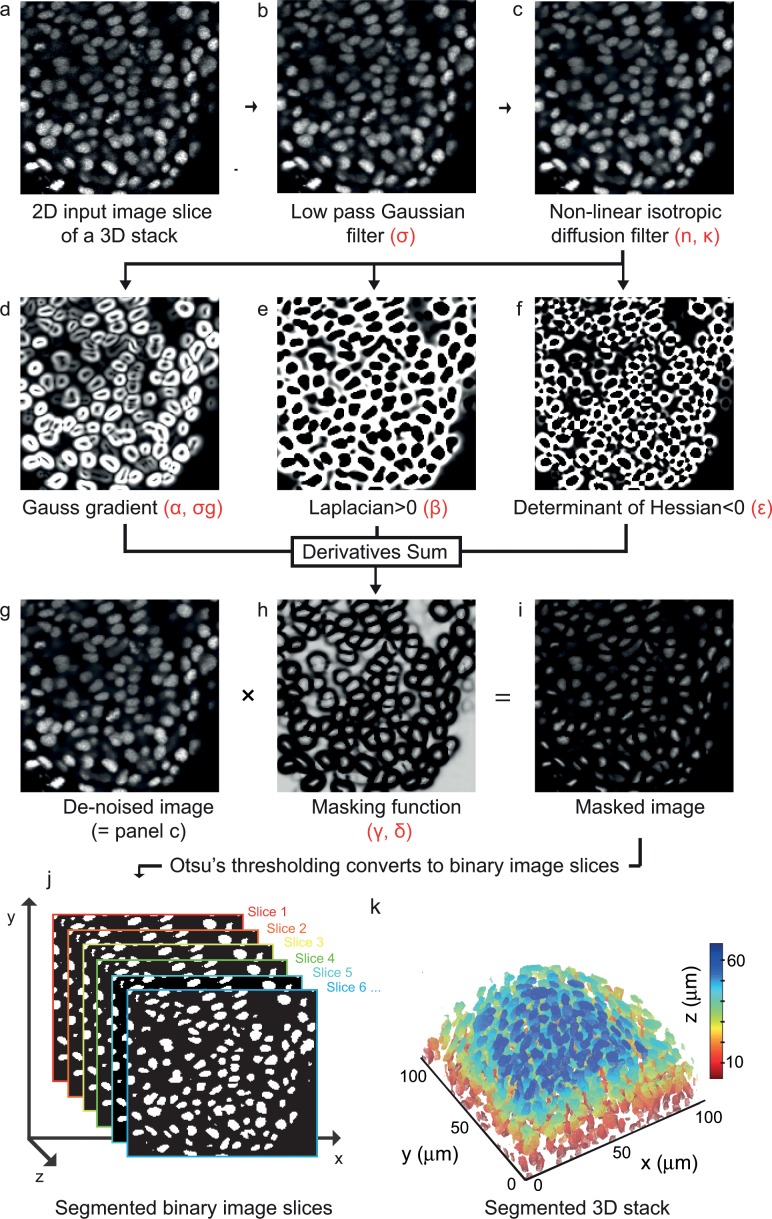
Derivatives sum algorithm for 3D nuclear segmentation. (a) 2D 8-bit gray scale image slice of a 3D stack of dimension 167 × 172 × 39 voxels from the posterior PSM of an 18-somite stage zebrafish embryo. (b) De-noised image after Gaussian blur (*σ =* 0.5, window size = 5×5 squared pixels). (c) Image smoothened by a non-linear isotropic diffusion filter (*κ* = 10, *n* = 4). (d) Magnitude of Gauss gradient (*σ*_*g*_ = 1.5), (e) Laplacian where positive and (f) Determinant of Hessian where negative, we show the absolute value. (g) De-noised image shown in (c). (h) A tangent hyperbolic masking function (*α = β = ε = γ = δ* = 1). (i) Masked image obtained by the pair-wise product between the de-noised image in (g) and the masking function in (h). (j) Slices of binary images obtained by Otsu’s thresholding method. (k) Surface rendered 3D binary objects colored with respect to their position along the *z*-direction. All tunable parameters are highlighted by Greek glyphs in red. See S1 Text for more details.

The raw datasets consisted of *z*-stacks of high-resolution 2D images taken from a zebrafish embryo in which the nuclei had been labeled with fluorescently tagged Histone2A protein (Fig 1A; [19]). Stacks were generated using a point scanning confocal microscope, and represent a single time point from time-lapse movies taken at a temporal resolution adequate for tracking of rapidly moving cells (Materials and Methods).

### The derivatives sum 3D nuclear segmentation algorithm

After acquiring the raw dataset, we began nuclear segmentation by smoothing the input images. Intensity fluctuations in the background and within nuclei in the raw images were first reduced by Gaussian filter (Fig 1B), with a σ chosen to preserve nuclear edges. Indeed, the segmentation protocol developed here largely depends upon strong gradients present at nuclear edges. The choice of de-noising filters and the subsequent steps aimed at preserving intensity variations at the edges and at the same time reducing the fluctuations in background and within the nuclei. In this example, we used a Gaussian filter, however depending upon the noise level and type in the acquired images, the choice of noise filters and their parameters varied. For images corrupted with a high level of noise, this initial filtering was complemented with median filtering or deconvolution. To further remove intensity fluctuations in the image nuclei whilst preserving nuclear edges, we applied a non-linear isotropic diffusion filter (Fig 1C; [20]). For further details, see S1 Text.

We computed the 2D spatial derivatives of images to obtain the Gauss gradient magnitude, the Laplacian, and the Hessian determinant. To specifically exploit the better resolution in *xy* than in *z* in classical confocal imaging, we restricted the computation to 2D, slice by slice. The nuclear edges appeared as broad maxima in the Gauss gradient magnitude image (Fig 1D). The positive Laplacian values (Fig 1E) enclosed the boundaries of nuclei. The negative determinant of the Hessian locates the saddle points (Fig 1F), i.e. the positions where two or more nuclear edges are touching each other. A key innovation in the proposed segmentation algorithm was to exploit these three derivative forms to formulate a 2D masking function (Fig 1H**)** whose steepness can be adjusted by specifying parameters. The entry-wise product of the masking function and the de-noised image produced a 2D-masked image (Fig 1I) with suppressed signal intensity at contacts between nuclei, thereby separating each nucleus.

Otsu's thresholding [21] was applied to each 2D-masked image slice of a 3D stack (Fig 1J). Foreground connected pixels from neighboring slices formed 3D binary objects of voxels (Fig 1K). By weighting the derivative parameters, it was possible to fine-tune the nuclear segmentation quality (S1 Fig). We will refer to the image segmentation protocol introduced so far as the “Derivatives Sum” algorithm (DS algorithm). Note that the segmented nuclei obtained with the DS algorithm might not exactly match the native morphologies and volumes due to the masking of nuclear boundaries. Nevertheless, the accuracy of the boundaries is sufficient to compare nuclear volumes between different embryonic regions or to quantify orientations of nuclei as a measure for cell packing.

### Post-processing of under-segmented objects in 3D

**Fig 2:**
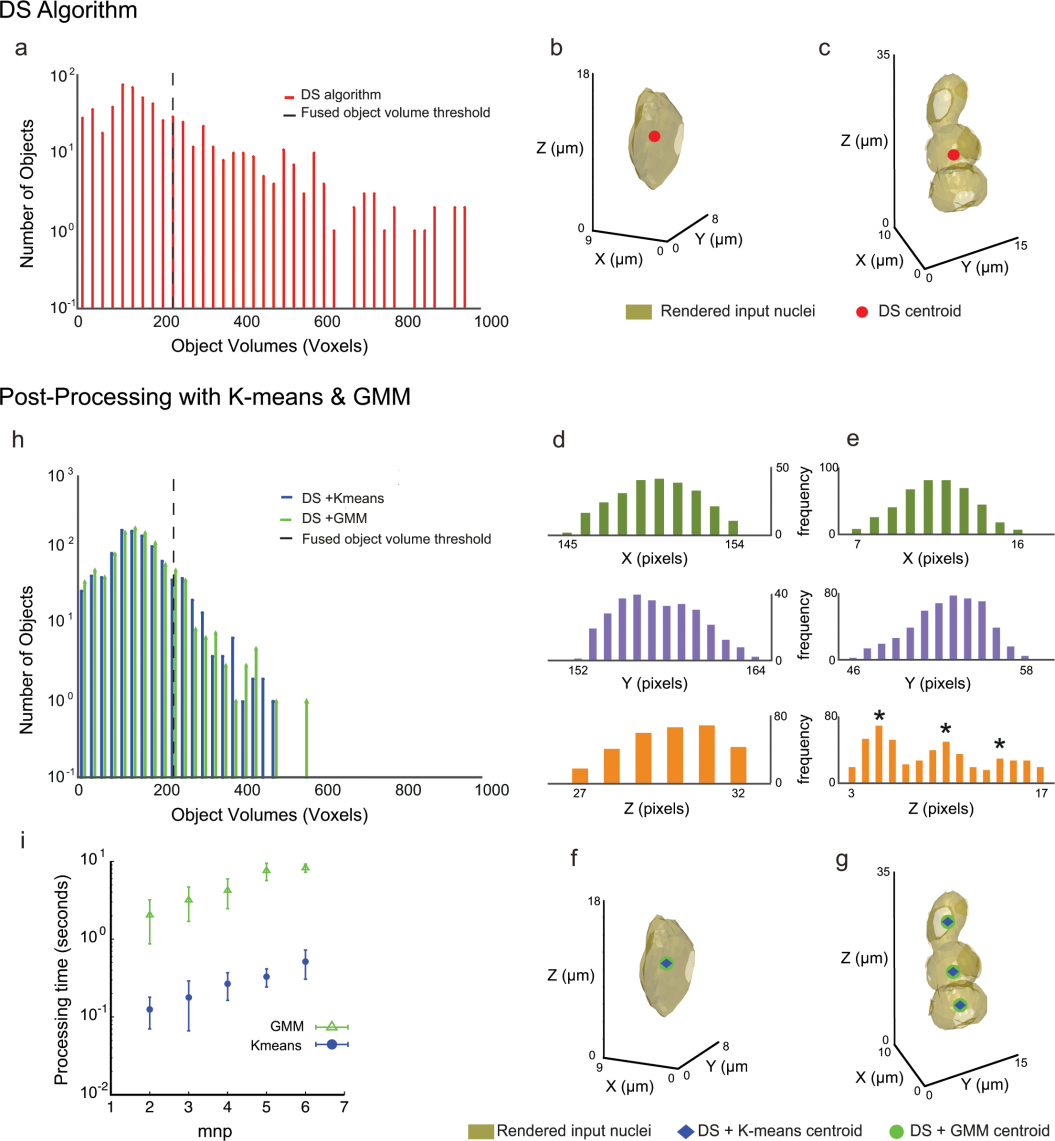
Post-processing steps to separate under-segmented objects. Top panel is a result of the DS algorithm and the bottom panel are the results obtained after post-processing steps for the single stack shown in Fig 1. (a) Stem plot of the volumes of segmented objects obtained after DS algorithm. The black dashed vertical line (at 237 voxels) indicates the empirically calculated ‘Fused object volume’ threshold value above which the objects (with volumes > 160 μm^3^) were subjected to post-processing steps. Position of the dashed line depends upon the DS algorithm and noise filter parameters and volumes of segmented objects (S1 Text). (b) A 3D rendered object from the left side of the black dashed line in (a) correctly segmented by the DS algorithm as indicated with the centroid position in red. (c) An example of 3D rendering of a fused object, as indicated by the position of the centroid in red, from the right side of the black dashed line in (a). (d) Frequency distribution of voxels in *x*, *y* and *z* direction for the correctly segmented nucleus in (b) obtained after DS algorithm. Note that a single nucleus has a unimodal distribution of voxels in each direction. (e) Frequency distribution of voxels in *x*, *y* and *z* direction for the fused case in (c). The distribution in the *z*-direction indicates 3 peaks marked by asterisks (*), suggesting three nuclei fused in the *z*-direction. (f) 3D rendered object shown in (b) is post-processed with K-means (centroid position in blue) and GMM (centroid position in green). All the three methods give the same centroid position. (g) 3D rendered object shown in (c) is post-processed with K-means and GMM that find 3 new centroid positions indicated in blue and green thus segregating fused nuclei. (h) Stem plot after DS with post-processing steps. Blue and green stem plots represent segmented volumes after DS with K-means and DS with GMM, respectively. Post-processing steps tend to reduce under-segmentation. (i) Dependence of the average processing time of post-processing steps GMM and K-means on the maximum number of local peaks *mnp*. Processing time was measured for each single object with a given *mnp* value. The error bars indicate standard deviations.

As a result of the DS algorithm, we obtained an extended distribution of object volumes (Fig 2A) and their centroids. Objects with volumes less than 10 voxels were considered as noise and ignored. The DS algorithm was considered to position the centroid correctly for single nuclear objects between 10 voxels and an empirically determined threshold value, termed the ‘fused object volume threshold’, computed as the mean volume of the segmented objects plus the standard deviation multiplied by a weight parameter *n* (S1 Text). The mean and standard deviation volume values depend on the parameters of the noise filters and the DS algorithm (black dashed line in Fig 2A; S1 Text). A typical example from the left side of the black dashed line in Fig 2A is shown in Fig 2B and an example from the right side is shown in Fig 2C. Like the latter example, objects to the right of the black dashed line frequently represent multiple fused nuclei, which were subjected to post-processing steps to re-segment and find the correct centroids and their respective volumes (S1 Text).

One way to fragment the fused objects is to heavily weight the parameter values contributing to the masking function in the DS algorithm, especially increasing the weight for the Gauss gradient magnitude (Fig 1D), such that the nuclear edges are eroded until fused nuclei are split (S1A Fig). This is particularly effective within high-density regions. However, this comes at the cost of decreasing nuclear volumes, especially within low-density regions where a fairly good segmentation is already achieved by not weighting the parameters heavily (S1B Fig).

To simultaneously reduce under-segmentation and preserve as much of the native morphology of the nucleus as possible, we compared the ability of two unsupervised clustering methods; K-means and Gaussian mixture models (GMM) [22–24] to locally post-process potentially fused nuclei from the set of large objects. These methods require *a priori* knowledge of the number of fused objects to split a cluster into candidate individual nuclei. The high variation in nuclear volumes for well-segmented nuclei in the image (Fig 2A) precluded the use of a mean nuclear volume to infer the number of nuclei fused as one object (i.e. number of fused objects = fused object volume/mean nuclear volume).

As the first step towards post-processing of fused candidates, we examined the frequency distribution of the voxel list in all the three directions to estimate the number of fused nuclei (Fig 2D and 2E; S1 Text). A correctly segmented nucleus had a unimodal voxel distribution in each *x*, *y* and *z* direction (Fig 2D**)**. In contrast, the voxel distribution of fused candidates had two or more peaks in some or all the directions (for 3 nuclei fused in the *z*-direction, peaks marked by asterisks in Fig 2E). Thus, the maximum number of local peaks (*mnp*, see S1 Text) in any of the directions (*x*, *y*, *z*) provided an estimate of the total number of fused objects.

Prior to implementing the clustering methods, the voxels were scaled in micron units (μm) (Figs 1K, 2B, 2C, 2F and 2G). K-means clustering was fed with the value of *mnp* to group the voxels based on their Euclidian distances such that they were segregated into potential individual nuclei (Fig 2G). These potential individual nuclei were again checked for a unimodal voxel distribution and for cases with *mnp* > 1 we re-iterated the K-means step to further segregate them into single candidate objects.

We initialized the GMM method with an over-estimated value of *mnp* (S1 Text). This generated a mixture of several Gaussians with different means and variances to fit the same voxel lists for each peak value that represented the number of possible fused nuclei. To determine the correct number of nuclei for the respective voxel list from the Gaussian parameters, we used the Akaike Information Criterion (AIC) [25] on the results obtained from GMM to find the best fitting Gaussian model. AIC determined the number of nuclei that best fit to the voxel data (Fig 2G). Thus GMM provided a powerful fitting tool, while the AIC helped to determine the best fitting parameters by preventing over-fitting.

We next confirmed that these methods of post-processing did not alter the centroid positions of individual nuclear objects that had already been correctly segmented by the DS algorithm (Fig 2F), ensuring that the local re-segmentation methods are not biased towards splitting nuclei. However, for the potentially fused object example in Fig 2C, both methods split the voxels into three and found their respective new centroids as shown in Fig 2G, consistent with the presence of three nuclei with touching boundaries, as confirmed visually. The number of objects with very large volumes considerably decreased after post-processing (Fig 2H). For the image stack analyzed in Figs 1 and 2, a total of 610 objects were segmented by DS and 210 were sent for post processing. K-means re-segments these into 663 objects and GMM re-segments them into 687 objects. This increases the number of objects from the under-segmented set approximately 3-fold, indicating the effectiveness of the K-means and GMM methods to rectify segmentation errors locally. The computational time for the stack size of 167 × 172 × 39 voxel dimensions for the DS method with K-means took 37 seconds whereas the DS algorithm with GMM processing took 596 seconds in Matlab (R2014b) on a 2,6 GHz Intel Core i5 processor. K-means is much faster than GMM in analyzing the same object (Fig 2I) since the latter builds models for the entire search space.

### Evaluation of segmentation protocol with synthetic images

**Fig 3:**
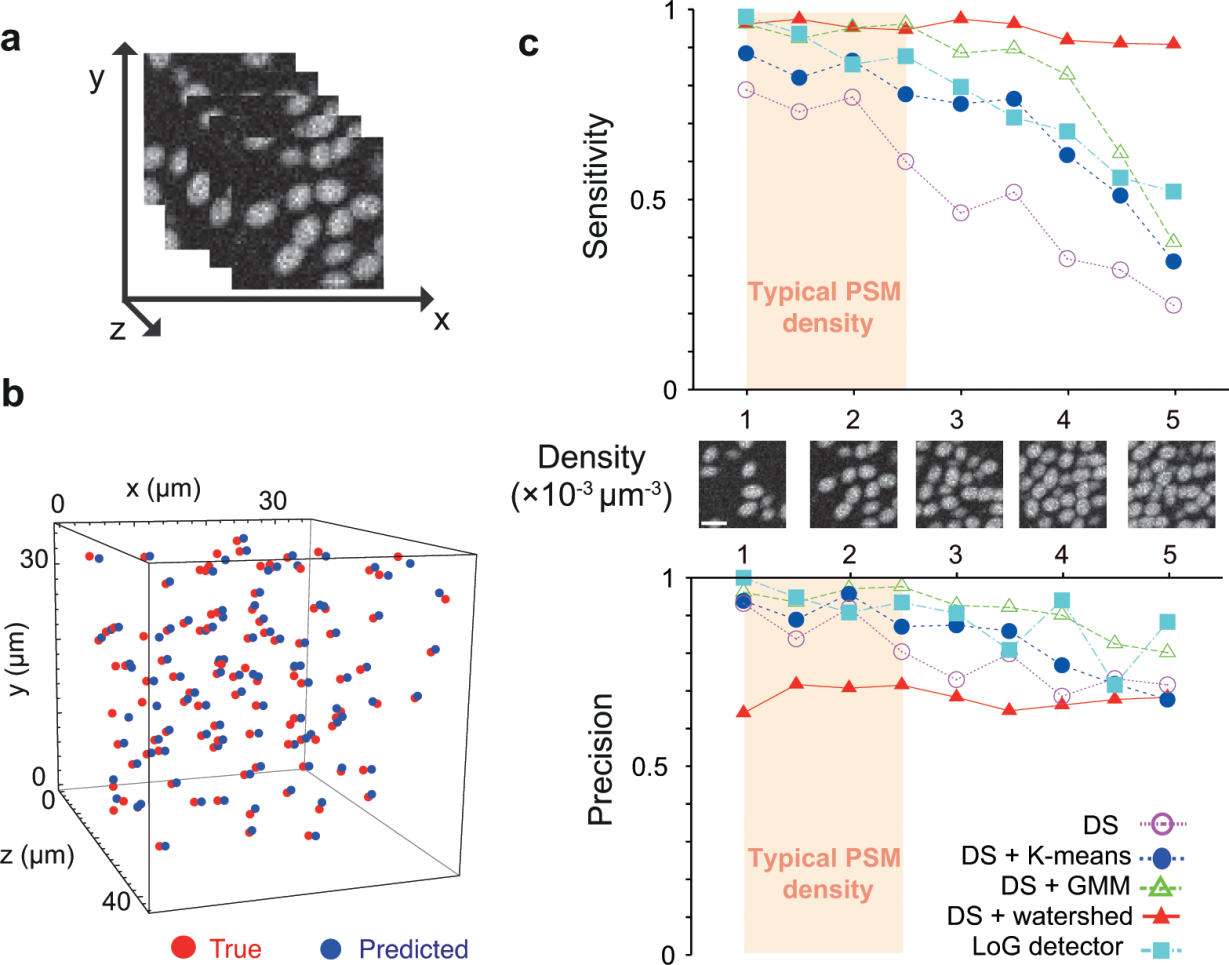
Assessment of 3D segmentation using synthetic image data. (a) A stack of slices of a representative synthetic image produced with characteristic SNR and object density. (b) Positions of true centroids of objects (red dots) and centroid positions detected by the Derivatives Sum algorithm (blue dots) in the synthetic image. (c) Dependence of sensitivity and precision of algorithms on the density of objects in synthetic images. SNR = 5. Gaussian filter (*σ =* 0.5, window size = 5×5 squared pixels) and median filter (window size = 3×3) were used here for de-noising. Parameters in DS are *α = β = ε = γ = δ =* 1, and *σ*_*g*_ *=* 1.1. Scale bars = 10 μm.

To systematically evaluate the performance of the DS algorithm and the post-processing steps, we generated synthetic 3D images in which the exact position of every object is known and where the image statistics can be tuned to match the experimental data (Fig 3A and 3B; [18]). Synthetic images have been previously used to evaluate segmentation algorithms for histopathologic tissues [26, 27]. Here we developed a model for the zebrafish PSM and tailbud nuclei based on our image acquisition settings. We modeled a cellular nucleus as an ellipsoidal object with parameters for nuclear density, background intensity and spatial resolution similar in value to those we measured in embryonic images. A gamma distribution function approximated the measured distribution of intensity fluctuations, both in nuclei and background. The Signal to Noise Ratio (SNR) was defined as the ratio of mean to standard deviation of the gamma distribution within a nucleus, which represents the strength of intensity fluctuations within a nucleus (for more details, see S1 Text).

We examined the efficiency of the DS algorithm by varying the density of objects in the synthetic images for SNR = 5, typical within the embryonic images. We determined the sensitivity, i.e., the detection rate of objects, and the precision, i.e., the probability that a detected object is a real object (Materials and Methods). The sensitivity of the DS algorithm was around 0.8 with density up 2×10^−3^ μm^–3^ but steeply decreased with further increase in density (open circles in Fig 3C). The result indicates that the DS algorithm itself tends to under-segment objects and is not robust for higher density values. This occurs because the frequency of touching objects in the *z*-direction increases with density, but the DS algorithm in its current instantiation does not use spatial derivatives along the *z*-direction of images, and therefore cannot separate touching nuclei in this direction. We also examined the dependence of sensitivity and precision on the parameters in the DS algorithm (S1A and S1B Fig). The weight of determinant of the Hessian *ε* increased sensitivity and precision without eroding object volumes, highlighting the importance of the Hessian in separating objects. The weight of the Gauss gradient *α* also increased sensitivity and precision but it strongly eroded object volumes, suggesting that weighting *α* heavily can be useful to segment densely packed nuclei. The weight of the Laplacian *β* only weakly affected the segmented object volumes.

Combining the DS algorithm with K-means and GMM post-processing improved sensitivity and precision. GMM (open triangles) outperforms K-means (closed circles) with increasing density values (Fig 3C).

To further evaluate GMM as a superior post-processing method, we compared these results to those obtained from the DS with the watershed algorithm as a post-processing step [28]. The watershed algorithm has been widely used to split fused nuclei [29]. Although post-processing with the watershed algorithm was highly sensitive, attaining about 0.9 regardless of density, its low precision, which was around 0.6 even for lower densities (Fig 3C), indicates that it severely over-segmented the objects. Precision is more important than sensitivity to avoid artifacts while addressing biological questions.

We also tested the common blob detector based on the Laplacian of the Gaussian (LoG, [30]) to detect only the 3D centroid positions of the objects in the synthetic images. The sensitivity and precision of the DS with GMM were better than those of the LoG detector (Fig 3C).

We examined the dependence of the above algorithms on SNR by changing SNR while fixing the density of objects constant (S2 Fig). We confirmed that the DS with GMM outcompeted the other algorithms listed above with different SNRs. The sensitivity and precision of the DS with GMM did not depend on SNR up to the density of 3×10^−3^ μm^–3^. As the density of objects was further increased (> 3×10^−3^ μm^–3^), the sensitivity of the DS with GMM decreased at lower SNR. This is because the number of contacts between objects increases with higher density, resulting in weaker gradients at the nuclear boundaries. In addition, stronger intensity fluctuations tend to destroy saddle points used by the DS algorithm and GMM for segmentation.

In some embryonic tissues orientations of nuclei may align in one direction due to cell packing. We tested the robustness of the DS algorithm with post-processing against changes in object orientations (S3 Fig). For this, the degree of alignment of ellipsoidal objects in the synthetic image was changed from random orientation to perfect alignment. We confirmed that the values of sensitivity and precision of the proposed algorithms depend very weakly on the orientations of objects (S3 Fig), suggesting that the algorithms can achieve a reasonable performance in different embryonic tissues with different nuclear alignment. Combined, these results showed the utility of synthetic data set to rapidly explore the performance of a segmentation protocol over a range of anticipated features of an image data set.

As we showed above, the GMM outperformed K-means across a range of object densities and SNRs with respect to the sensitivity and precision. This is because K-means simply clusters the voxels based on the maximum number of local peaks *mnp* in the voxel frequency distribution. However, such a distribution may also capture unfiltered local noise fluctuations leading to wrong estimates for number of nuclei in some cases. In contrast, GMM explores several possible models, which in combination with AIC tends to find the correct number of nuclei that fits to the voxel data. Because GMM explores different models within a search space criterion (S1 Text), it needs computational time an order of magnitude longer than K-means for the same *mnp* case (Fig 2I). If the density of objects is sparse and SNR is high, the sensitivity and precision of K-means are comparable with those of GMM (Fig 3C and S2 Fig), and the computational time for K-means is shorter than that of GMM. In this situation, K-means outperforms GMM.

### Chimeric embryos provide ground truth benchmark for experimental data

**Fig 4:**
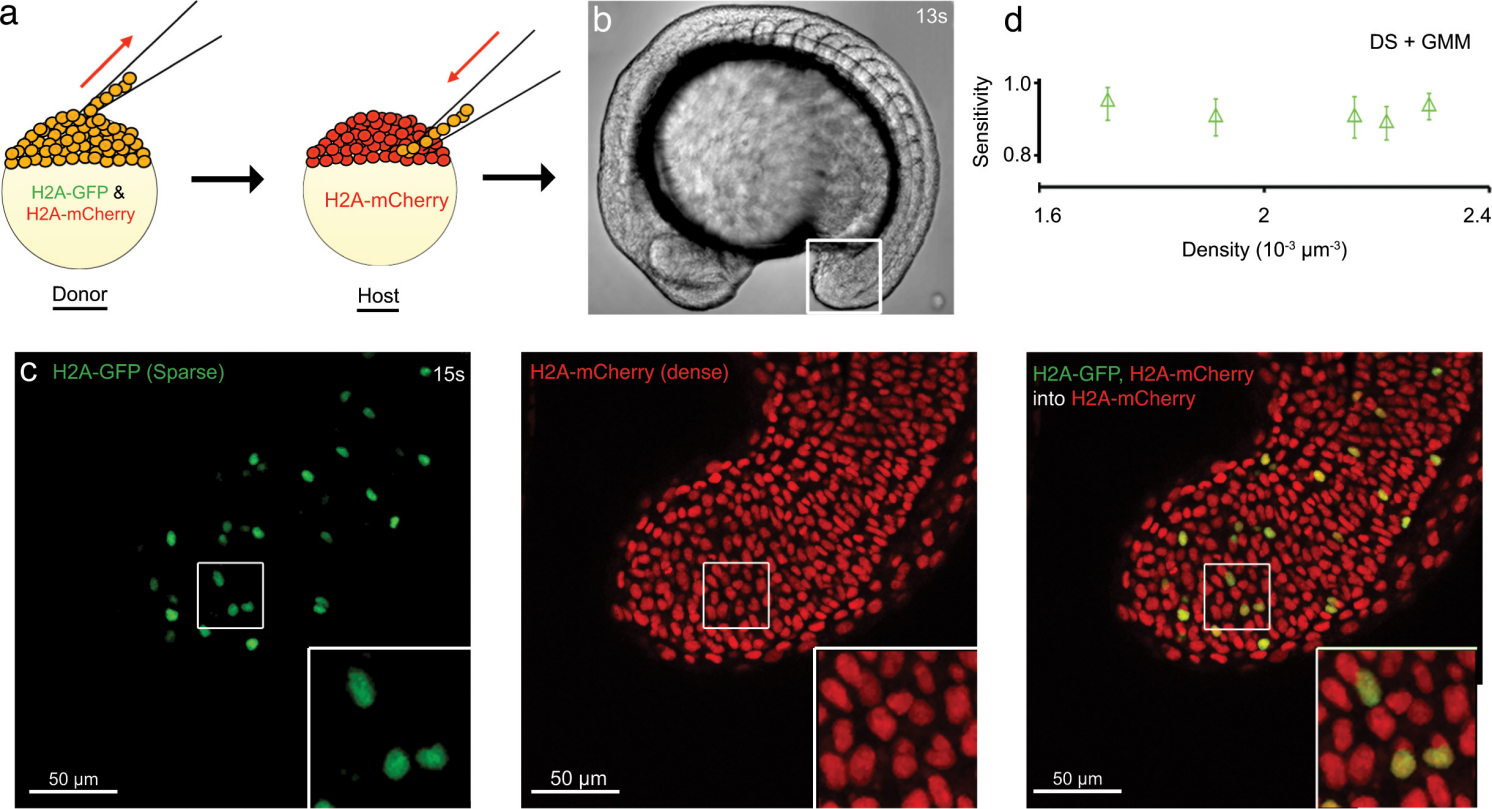
Assessment of 3D segmentation using experimental image data. (a) Schematic illustration of transplantation experiment. Cells in a donor embryo at blastula stage expressing two histone variants fused to GFP and mCherry (*h2Aflv-gfp*/ *h2Aflv-mCherry*) are colored orange. (b) Bright-field image of a 13 somite-stage chimeric embryo. The white box indicates the tailbud. (c) 15 somite-stage chimeric embryo in gfp (left) and mcherry (middle) channels, and merged (right). White box in each image indicates the cropped region (50×50 squared pixels) used for the sensitivity analysis. Inset image is magnification of boxed region. (d) Sensitivity plot over density for five cropped images from four chimeric embryos. Each symbol and error bar indicates the temporal average and standard deviation, respectively, of the sensitivity over 10 time frames for a cropped image. Parameters in DS are *α = β = γ = δ* = 1, *ε =* 2, and *σ*_*g*_ *=* 1.2. De-noising filters; Gaussian filter (*σ =* 0.5, window size = 5×5 squared pixels) and median filter (window size = 3×3), Lucy-Richardson deconvolution filter with *σ =* 0.5, non-linear isotropic diffusion filter (*κ =* 50, *n =* 5). Both channels for all five cropped images were processed with same parameter values.

It is extremely difficult to obtain ground truth data sets for benchmarking segmentation algorithms on embryonic images. Manual segmentation is only feasible for small data sets. Consequently, it would be valuable to have a method to generate an internal ground truth for large embryonic data sets. In a previous study, nuclei in zebrafish tissues were double-labeled by micro-injection of RFP and GFP mRNAs and accuracy of cell tracking was evaluated by comparing these two channels [31]. To generate stable double-labeled nuclei, we constructed embryo chimeras by transplanting 20–30 cells from blastula stage embryos that express histone variants fused to both GFP (*h2AflV-gfp*; green nuclei) and mCherry (*h2AflV-mCherry*; red nuclei) into age-matched host embryos that expressed only *h2AflV-mCherry* (Fig 4A). The resulting chimeric embryos had a small number of nuclei co-labeled with mCherry and GFP at a sparse density within the high-density, mCherry-labeled total nuclear population (Fig 4C). Sparsely distributed nuclei expressing GFP are straightforward to segment by almost any means because they very rarely touch their neighbors. The known positions of these nuclei can be compared to the predicted positions of nuclei expressing mCherry at a high density as determined by any segmentation algorithm. This experiment provides a natural image dataset where a nuclear segmentation algorithm can be verified by use of the sparse channel that forms the ground truth and the dense channel that forms the test set.

We made time-lapse movies of the tailbud and PSM of chimeric embryos during axis extension (13–20 somite stages; Fig 4B), because the tissue contains a range of nuclear densities, shapes and movements in 3D. These movies consisted of *z*-stacks as previously described, which were acquired at frame rates high enough to follow nuclear movements. To reduce the computational cost of analyzing properties such as nuclear density and SNR, we selected small cubic regions of approximately 35×35×61 μm^3^ with different nuclear densities (Fig 4C). We segmented nuclei in both dense and sparse channels using the DS with GMM. We visually confirmed the accuracy of the segmentation results for the sparse channel, which was 97% on average. Rare cases of mis-segmentation resulted from touching nuclei due to recent cell division. Thus, the positions of the nuclei from the sparse channel appeared suitable for use as a ground truth data set.

We investigated the sensitivity of the algorithm in the dense channel against the ground truth data set from the sparse channel. We analyzed five cubic regions differing in nuclear densities in both the sparse and dense channel and examined the centroid positions match between the two channels (Materials and Methods). The measured sensitivity over a density range from 1.6×10^−3^ to 2.4×10^−3^ μm^-3^ was around 0.93 (Fig 4D), a performance as good as the one measured on synthetic data. This consistency also supports the robustness of evaluation by synthetic images. The temporal standard deviation of sensitivity over time frames is small, ~ 0.05 (Fig 4D and S4 Fig), demonstrating the robustness of our method to image fluctuations. In S4 Fig we examined the dependence of sensitivity on the volume-threshold parameter *n* for post-processing (S1 Text). When the value of the parameter *n* is smaller, the fused object volume threshold, FOV (black dashed vertical line in Fig 2A) becomes smaller and more objects are post-processed. Here, for a densely packed tissue, post-processing more objects increased sensitivity (S4 Fig). If the tissue packing is less dense, and nuclei are well separated naturally, one may choose a higher value for *n* that will result in fewer objects sent for post-processing, thus decreasing the computational time, while maintaining the segmentation accuracy.

Taken together, we have demonstrated that sparse-dense dual-labeled chimeric embryos can be used to create a ground truth dataset within the context of living embryos that can be used to benchmark the efficiency of segmentation algorithms. The same technique may be further extended to verify nuclear tracking algorithms. Our analysis confirms that the DS with GMM method accurately and robustly segments densely packed nuclei in living tissues.

## Discussion

### Iteration of imaging, image processing and benchmarking

Here we propose a three-step procedure for imaging, image processing and benchmarking that can be applied to many complex and challenging biological image problems. We provide a novel algorithm to segment densely packed nuclei, a method to test segmentation algorithms on synthetic image data, and an experimental protocol to produce a ground truth data set that can be used to accurately measure the performance of an algorithm on real-world data. Depending on the sample to be imaged, other segmentation algorithms may prove to be more appropriate, but the three-step procedure should allow an iterative improvement in imaging and image processing, leading to a higher quality analysis.

The DS algorithm exploits better resolution in *x* and *y* relative to the *z* direction to build a crown-like mask that helps separate touching nuclei. The DS algorithm takes advantage of the high definition to smooth the nuclear interior before determining the gradient properties. We used the features of 3D segmented objects for post-processing steps that further refined the segmentation results. Our method is not limited to measuring nuclear positions, but can also segment the entire nucleus. It is therefore applicable in studies that aim to follow subcellular structures over time in single cells, or within large, moving and densely packed tissues, or in studies following the cell or nuclear shape.

Various segmentation algorithms have been previously proposed to deal with densely packed nuclei. Algorithms used previously for nuclear segmentation include LoG and DoG blob detectors [32, 33] that are well-established methods and can be easily parameterized for the detection of spherical objects. However, such methods rely on finding the intensity maxima and assume that the nuclear volumes have similar sizes. From our analysis, the PSM and tailbud nuclei vary in size and shape wherein the epithelial nuclei are larger than the PSM nuclei and as we image deeper into the tissue close to the central nervous system, the nuclei are comparatively small and elongated. The multiscale LoG with automatic scale selection greatly improves upon the fixed-scale method, however is inadequate for heterogeneous clusters of nuclei with different sizes, and weak separating edges [34]. This makes blob detector based algorithms difficult to use for our tissue of interest. Kofahi and co-workers [34] have used the LoG filter only for seed detection followed by graph-based coloring method for segmentation of images.

A method based on the diffusion gradient vector field, whose solution to the partial differential equation (PDE) describes the deformation of an elastic sheet, has been used to find nuclear boundaries for zebrafish PSM nuclei [35]. A local adaptive thresholding method was used thereafter to segment the 3D objects. Although the method seems to achieve good results, PDE-based methods typically need good computational hardware and require the stopping and re-initialization criterion during the evolution of the PDE to be set in advance in order to obtain a smooth curve along the nucleus. In this work, we computed the image gradients and second derivatives tunable with suitable parameters in a single step. Such an implementation is fast and achieves a first level segmentation in 3D that is further refined to obtain better accuracy by subsequent post-processing.

Amat et al. proposed the concept of supervoxels [29], which is similar to the voxel list we have proposed in this work. However, we generated the voxel list (where a 3D nucleus is defined with a set of connected voxels) using the DS algorithm and only split the fused nuclei with consecutive post-processing steps. The supervoxels [29] are constructed using a watershed algorithm that tends to over-split nuclei, however this problem is overcome by using a persistence-based clustering method to split and merge the supervoxels. Our method, in its current instantiation, does not over-split the nuclei. As a secondary step, to cluster a few supervoxels in space (segmentation) and time (tracking), Amat et al. used a Bayesian approach with GMM, which is similar to our post-processing implementation (GMM with AIC) to achieve segmentation alone.

We introduced here, for the first time, the concept of the frequency distribution in the voxel lists as an initial cue to estimate the number of clusters that are fused together. By knowing a minimal number of fused clusters based on the initial cue, our GMM method saves computational time. Furthermore, by introducing an empirical threshold (FOV) for post-processing (Fig 2A), our algorithm provides adjustability and flexibility between computational time and segmentation accuracy (S4 Fig).

The post-processing steps implemented to separate objects that remain fused after the operation of the DS algorithm resembles a “divide and conquer” strategy (Gene Meyers, personal communication). Instead of designing a single, potentially complex and computationally expensive algorithm that attempts segmentation of the entire data set in one step, we have applied a sequence of relatively simple steps to tackle smaller yet successively more difficult portions of the data. This has the advantage of making the process more modular and restricting computationally expensive steps to small data sets. Although our algorithm performs well on images of zebrafish tissue, a detailed comparison of segmentation performance between different algorithms will be an important piece of work in the field of developmental biological image processing.

Modern quantitative imaging techniques and tools require appropriate benchmarking, a particular challenge *in vivo*. In this paper, we have proposed benchmarking methods using synthetic images and experimental data from embryo chimeras. Synthetic images allow quantitative evaluation of any segmentation algorithm and quickly reveal its advantages and shortcomings with respect to testable features of real world images. This rapid comparison allows tuning of image processing parameters to optimize the performance of the algorithm(s) against a range of likely target image qualities. Conversely, the performance of an algorithm on synthetic data can be used to suggest ways of optimizing the imaging parameters. Interestingly, we find the performance of the DS with GMM method depends modestly on SNR within the range of densities observed in live tissue (S2 Fig). This is a desirable property, as it grants a certain freedom when imaging the specimen, and allows lower excitation power, thereby reducing phototoxicity [36]. The robustness to SNR changes is in part a consequence of the high definition of nuclei in the images.

To generate the *in vivo* test data set to serve as the ground truth for evaluating image-processing performance, we used sparse-dense dual-labeled chimeric embryos. This method to generate a ground truth data set is applicable in many species, including chick, mouse, fruit fly and others, where sparse-dense dual-label chimerism can be induced by genetic or embryological manipulation.

### Relevance of image segmentation accuracy to biological questions

By combining the DS algorithm with GMM we achieve an accuracy of ~90% on both synthetic data and real images of nuclei in a tissue. Whether this accuracy is high enough can only be answered with respect to the specific biological application. For some questions, such as determining nuclear density, shape and orientation, it is sufficient to estimate the ensemble properties of the detected objects in the tissue, and small errors in segmentation are likely not to affect the conclusions. Our processing strategy will perform well at these tasks. For questions that require knowledge of short- to medium-term cellular movements obtained by tracking, such as determining the rate of cell mixing with their neighbors, or the local flow of cells, the segmentation strategy should also perform well. For applications where the individual identity of a cell is critical, such as long-term fate mapping, segmentation accuracy must aim to be perfect, and manual annotation will be necessary. Importantly, as long as the accuracy of a segmentation algorithm can be reliably estimated on appropriate synthetic and ground truth data sets, an informed decision can be taken about whether the accuracy is high enough for the question at hand.

## Conclusion

The proposed DS segmentation algorithm combined with GMM and AIC achieves a reasonably good accuracy in the zebrafish tissues. The algorithm provides flexibility to optimize accuracy and computational time depending upon the *in vivo* nuclei features such as size, density and SNR. Testing the usefulness of this method for images of other tissues and for those acquired with different microscopes [3, 29] would be an important future work. Synthetic and chimeric embryonic images allow systematic evaluation of the segmentation algorithm and its parameters. Thus, an iterative procedure for imaging, image processing and benchmarking may lead to reliable quantification of single cell behavior analysis in developmental biology.

## Supporting Information

S1 Appendix Matlab scripts for image segmentation algorithm. The Matlab codes for the segmentation pipeline presented in the paper.
(ZIP)

S2 Appendix Generation of synthetic images: C code and Mathematica. The Mathematica and C code for the generation of synthetic images presented in the paper.
(ZIP)

S1 Dataset Example image data of a zebrafish embryo. A cropped region from a 3D stack confocal image (tif format) of the PSM tissue of an 18-somite stage developing zebrafish embryo analyzed in Figs 1 and 2 in the main text.
(ZIP)

S1 Fig Dependence of the derivatives sum method on the weighting of parameters for Gauss gradient *α*, Laplacian *β* and determinant of Hessian *ε* in synthetic images. For each column of panels, only one parameter is varied while the others are fixed at a value of 1. For example, in the first column *α* varies and *β* = *ε* = 1. (a) Sensitivity and precision of segmentation using DS algorithm as a function of the object density in the synthetic images. (b) Dependence of the mode of the volume histogram for the segmented objects on *α* (left), *β* (middle) and *ε* (right). Examples of volume histograms for selected values of parameters are shown as insets. No post-processing methods were used here. SNR = 5. Object density in (b) was 2.5×10^−3^ μm^–3^. See also S1 Text.
(EPS)

S2 Fig Segmentation algorithms performance comparison with different signal-to-noise ratios (SNRs) and object densities. Sensitivity and precision were computed for synthetic images. In each panel SNR varies for a constant object density. The top row shows sensitivity and the bottom row shows precision. Parameters in the DS method are *α = β = ε = γ = δ* = 1.
(EPS)

S3 Fig Dependence of sensitivity and precision on orientations of objects in synthetic images. Sensitivity and precision were computed for synthetic images where the degree of alignment of ellipsoidal objects was varied. The degree of alignment was defined as the largest eigenvalue of the nematic tensor for the long axis of objects, Q_*αβ*_ = (3/2*N*)**Σ***u*_*iα*_*u*_*iβ*_ *–*(*δ*_*αβ*_ /2) where *u*_*i*_ is the unit vector pointing the direction of the long axis of object *i* (*α*, *β* = *x*, *y*, *z*), *N* is the total number of objects and *δ* is Kronecker delta function. Smaller values of the horizontal axis indicate that orientations of objects are more random. The degree of alignment of objects in Fig 3 in the main text was about 0.67. SNR = 5. Density of objects was fixed as 2.0 × 10^−3^ μm^–3^. 3D centroid positions of objects were same in all the three orientations. Parameters in the DS algorithm were *α = β = ε = γ = δ* = 1. The scale bar represents 10 μm.
(EPS)

S4 Fig Dependence of sensitivity on the empirical value *n* affecting fused object volume threshold for post-processing in a single chimeric embryo. (a) Α 16-bit gray scale image cubic region of approximately 35×35×61 μm^3^ of a 16-somite stage zebrafish embryo was analyzed here, also in Fig 4. Sensitivity values across 10 consecutive time frames for different values of *n* that affects threshold for the fused object volume FOV (see S1 Text). GMM was used as a post-processing method after the DS method. Higher values of *n* shifts the FOV line more to the right on the volume plot in Fig 2A resulting in fewer objects sent for post-processing, with decrease in algorithm computational time, however reducing segmentation accuracy. (b) The average sensitivity and standard deviation over 10 consecutive time frames plotted against the volume-threshold parameter *n*. Parameters in DS are *α* = *β* = *γ* = *δ* = 1, ε = 2, and *σ*_g_ = 1.2. See main text for definition of sensitivity in chimeric embryo analysis.
(EPS)

S1 Text Descriptions of: (1) the derivatives sum algorithm and post-processing steps with K-means and GMM and (2) synthetic data generation. (DOCX)
