# Correction: Object Segmentation and Ground Truth in 3D Embryonic Imaging

**DOI:** 10.1371/journal.pone.0161550

**Published:** 2016-08-16

**Authors:** Rajasekaran Bhavna, Koichiro Uriu, Guillaume Valentin, Jean-Yves Tinevez, Andrew C. Oates

The first author’s name appears incorrectly in the published article. The correct name is: Rajasekaran Bhavna. The correct citation is: Bhavna R, Uriu K, Valentin G, Tinevez J-Y, Oates AC (2016) Object Segmentation and Ground Truth in 3D Embryonic Imaging. PLoS ONE 11(6): e0150853. doi:10.1371/journal.pone.0150853
