# Supplementary material for: Object Segmentation and Ground Truth in 3D Embryonic Imaging
Source: PLoS One. 2016 Jun 22;11(6):e0150853. doi: 10.1371/journal.pone.0150853 (PMC4917178; doi:10.1371/journal.pone.0150853)
Supplement: S1 Text — (DOCX) [file pone.0150853.s008.docx]

Object segmentation and ground truth in 3D embryonic imaging

Bhavna Rajasekaran^1,2^, Koichiro Uriu^1,2,3,4^, Guillaume Valentin^1,5,6^, Jean-Yves Tinevez^1,7^, Andrew C. Oates^1,5,6*^

1 Max Planck Institute of Molecular Cell Biology and Genetics, Dresden, Germany

2 Max Planck Institute for the Physics of Complex Systems, Dresden, Germany

3 Theoretical Biology Laboratory, RIKEN, Saitama, Japan

4 Graduate School of Natural Science and Technology, Kanazawa University, Kanazawa, Japan

5 Francis Crick Institute, Mill Hill Laboratory, London UK

6 Department of Cell and Developmental Biology, University College London,

London UK

7 Imagopole/CiTech, Institut Pasteur, Paris, France

* Corresponding author

E-mail: andrew.oates@crick.ac.uk

# S1 Text

Here we describe the mathematical details of the derivatives sum (DS) segmentation algorithm (section 1) and producing synthetic images (section 2). The reference list for this supplementary text is at the end of this note.

## 1. Nuclear segmentation protocol

### 1.1. Input image de-noising

Image de-noising is a crucial step in image analysis. This smoothens out the noise between pixels and at the same time does not degrade features of interest in the image. Since the algorithm relies on sharp nuclear edges, we prefer to de-noise images such that we dilute the noisy component without degrading the change of image gradients at the edges. We used linear or non-linear filters depending upon the noise quality present in the images [1, 2]. These filters include Median, Wiener, Gaussian [3] or for some cases Bilateral filter and Lucy Richardson Deconvolution filter [4, 5].

We used a Gaussian filter given by

$$g\left(x,y;\sigma\right)=\left(1/2\pi\sigma^{2}\right)\exp\left[-\left(x^{2}+y^{2}\right)/2\sigma^{2}\right]$$

to filter out low background noise in images. For images with high noise, we first used the Gaussian filter followed by non-linear filters. After applying the linear/non-linear filters, we obtained a de-noised image *IM*(*x*, *y*) where *x* and *y* denotes the position of pixels in a 2D slice of an image.

### 1.1.1. Non-linear isotropic diffusion filter

Originally proposed by Perona and Malik [6], the non-linear isotropic diffusion filter evolves the image under a smooth partial differential equation, similar to the heat equation. It yields intra-region smoothness while impeding diffusion at the edges. We applied the filter on the de-noised image *IM*, implementing the standard filter as:

$\frac{\partial IM(x,y)}{\partial t}=\nabla\cdot\left\{D_{f}\left(\left|\nabla IM\right|\right)\nabla IM\right\},$ (S1)

where $\left|\nabla IM\right|$ is the modulus of the de-noised image intensity gradient and *D_f_* is the diffusion co-efficient given by

$D_{f}\left(\left|\nabla IM\right|\right)=\exp\left(-\left|\nabla IM\right|/\kappa^{2}\right)$.

### 1.2. Computation of image derivatives

### 1.2.1. Image Gauss gradient: first derivative of the image

After image de-noising, edge pixels in the image were enhanced compared to the background and pixels within the nucleus. We convolved the de-noised image *IM* with the derivative of Gaussian *g*(*x*,*y*;*σ_g_*) to obtain the intensity gradient $\nabla IM\left(x,y\right)=\left[\partial_{x}g*IM,\partial_{y}g*IM\right]$,

where * denotes convolution. Then, we computed the magnitude of the Gauss gradient image $\left|\nabla IM(x,y)\right|$.

### 1.2.2. Image Laplacian and Hessian: second derivative of the image

By computing the second derivative of the Gauss gradient image, we obtained the Hessian matrix at each pixel:

$H_{m}\left(x,y\right)=\left(\begin{matrix} \frac{\partial^{2}IM}{\partial x^{2}} & \frac{\partial^{2}IM}{\partial xy} \\ \frac{\partial^{2}IM}{\partial xy} & \frac{\partial^{2}IM}{\partial y^{2}} \end{matrix}\right).$ (S2)

The trace of the matrix in (S2) is the Laplacian of the image $L_{a}\left(x,y\right)=\partial^{2}IM/\partial x^{2}+\partial^{2}IM/\partial y^{2}$.

For the masking function introduced below, we define the following two functions:

$L\left(x,y\right)=\left\{\begin{matrix} L_{a}(x,y) \\ 0 \end{matrix}\right.\begin{matrix} L_{a}(x,y)>0 \\ L_{a}(x,y)\leq0 \end{matrix},$ (S3)

and

$H\left(x,y\right)=\left\{\begin{matrix} \det H_{m}\left(x,y\right) \\ 0 \end{matrix}\right.\begin{matrix} \det H_{m}<0 \\ \det H_{m}\geq0 \end{matrix}.$ (S4)

*H*(*x*, *y*) highlights all the saddle points in the image. This component efficiently cuts through the touching spaces between neighbouring nuclei, thus contributing to the robustness of segmentation results (Fig 1f and S1a Fig). The positive Laplacian marks the outer nuclear edges.

### 1.2.3. Combining derivatives for the masking function

Adding the magnitude of Gauss gradient, positive Laplacian (S3) and the negative determinant of Hessian (S4), with different weighting constants *α*, *β*, and *ε* respectively, we obtain *DS* (*x*, *y*):

$DS\left(x,y\right)=\alpha\left|\nabla IM(x,y)\right|+\beta L(x,y)+\varepsilon H(x,y).$ (S5)

In order to obtain a smooth edge for every object, we used a tangent hyperbolic function that allows us to control the slope of the edge. Combining the derivatives with the tangent hyperbolic function, we defined a masking function *F*, such that it is zero at the edges and touching points of nuclei, while it is almost one in background and intra-nuclear spaces:

$F\left(x,y\right)=\frac{1}{2}\left\{\tanh\left(\gamma-\frac{DS\left(x,y\right)}{\delta}\right)+1\right\},$ (S6)

where *γ* and *δ* are parameters (Fig 1h). The parameters *α*, *β*, *ε*, *γ* and *δ* may be weighted suitably to achieve good segmentation results. S1 Fig shows the dependence of the algorithm on *α*, *β*, and *ε*.

The Hadamard product of the masking function *F*(*x*, *y*) and de-noised image *IM*(*x*, *y*) results in the final image *FI*(*x*, *y*) (Fig 1i).

### 1.3. Image thresholding

We used Otsu’s method [7] for thresholding each slice in *FI*(*x*, *y*) that was assembled with emphasized nuclear edges (Fig 1j). The threshold value depended on the variation in contrast and nuclear density.

### 1.4. Connecting similar pixels: 2D to 3D

2D binary slices are connected such that nearest neighbor foreground pixels (6 neighbors in a 3D stack) are clustered together as objects (Fig 1k; [8]). Each 3D segmented object is characterized by: (i) voxel list, the voxel coordinates in *x*, *y* and *z*, (ii) centroid, the geometric mean of clustered voxels, and (iii) volume, the total number of voxels.

### 1.5. Post-processing based on properties of 3D segmented nuclei

The imaging volume spans over different regions of the developing embryonic tissue. Therefore a wide range of heterogeneous shapes and sizes of nuclear volumes is observed even for correctly segmented objects. However, multiple fused objects incorrectly identified as a single object tend to have very large volumes. We empirically determined a threshold for the fused object volume *FOV*, above which all objects are considered for post-processing. For a 3D stack, the threshold value is automatically computed, and is influenced by the choice and parameters of the noise filters and the DS algorithm and the range of segmented volumes. The threshold is calculated based on the mean volume *MV* and the standard deviation of volumes of segmented objects *SDV*:

$FOV=MV+n\cdot SDV.$ (S7)

We examined a range of values of *n* (S4 Fig) and found that *n* = 0.1 provided a reasonable value of *FOV*. Each candidate fused 3D object was subjected to a post-processing step that locally operates to correct potential under-segmentation. We first sampled the voxel list in *x*, *y* and *z* directions. We counted the number of local peaks in the voxel list distributions and considered that the maximum number of the local peaks (*mnp*) among the *x*, *y* and *z* directions represents the total number of potentially fused objects. For a single correctly segmented object, *mnp* = 1 (Fig 2d), whereas for fused objects *mnp* > 1 (Fig 2e). The *mnp* value was fed to the K-means clustering algorithm that segregated the objects into *mnp* clusters of voxels. K-means clustering uses the Euclidian distance metric and the variance between the data points to determine cluster centroids [9-11]. It is vital to reduce local noise from the image, while keeping smooth intra-nuclear regions, to avoid *mnp* values misrepresenting the number of fused objects. K-means was applied with 2 iterations. In the second iteration we again looked for a unimodal distribution of voxels and for cases where *mnp* >1, the clusters were re-segmented using K-means.

Alternatively, Gaussian mixture models (GMM) [11, 12] were used for exploring a number of parametric models, one of which would appropriately fit to the voxel list data. The *mnp* value may under-estimate the number of fused objects present in the voxel list data. Therefore, we explored parametric models up to (*mnp+*4*)* to determine the correct number of Gaussian models. The Akaike information criterion (AIC) [13] was implemented at this stage, which automatically determined the number of Gaussian models, i.e. nuclei, that best fit to the voxel list data with smallest number of model parameters.

### 1.6. Detailed criteria for determining *mnp* value for post-processing from image resolution

The number of voxels comprising a nucleus/object depends on the voxel size during image acquisition. We imaged nuclei with voxel size of 0.691 × 0.691 × 1.75 μm^3^ in the *x*, *y* and *z* directions, respectively. For this image resolution, the smallest size of a single nucleus was 3 μm in the *x* and *y* directions (corresponding to 4 pixels) and 1 μm in the *z*-direction (corresponding to one pixel). A typical voxel distribution of a single nucleus shows single peak around the center in each direction. Cases representing fused nuclei have a greater size and multiple peaks in some or all the directions. We then use the number of peaks in the voxel frequency distribution to determine the number of fused nuclei, choosing *mnp* as the maximum number of peaks among all directions. For this, peaks are not counted if their distance in *x*, *y* is less than 4 pixels, which would be less than the minimal nuclear size for the image resolution used here.

### 1.7. Selection of noise filter parameters and DS algorithm parameters

The choice of the noise filters and their parameters for real image data was made by visual inspection. Because the DS algorithm tunes the edges of nuclei, it is pertinent to smooth inter-nuclear regions, while at the same time not diffuse information between touching boundaries of nuclei. This may be visually verified for a few slices and then can be applied for the entire stack and even over consecutive time points.

The parameters of the DS algorithm are tuned such that the nuclear edges are marked well in the 2D slices. Again, at this step visual inspection is required. We provide a Matlab code that uses standard in-built commands for visual inspection at different steps of the algorithm. In confocal images, with high density of nuclei, it is not possible to obtain high segmentation accuracy at this stage. The stem plot of volume distribution (Fig 2a) can give insight on the segmentation results.

We provide the Fiji implementation of the DS algorithm with noise filters (Gaussian, median and non-linear diffusion) using a Graphical User Interface. At every step of the DS algorithm, which is weighted with a parameter, it is possible to vary parameter values with a slider in the interface that at the same time visually updates the images. In this way, Fiji provides users a better idea as what set of DS algorithm parameters need to be used for their images.

Because statistics of real image data vary depending on how data is acquired, it is not possible to provide one set of parameters as to be correct. For our embryonic images, we used de-noising filters depending upon the image quality. We further tuned parameters of the DS algorithm to obtain better separation at the nuclear edges by visual inspection. For the post-processing steps, we provide Matlab code to visualize segmentation results using 3D rendering of voxels and centroids (Figs 2b, 2c, 2f and 2g). To achieve a better understanding of the 3D image data and segmentation strategies, we strongly recommend the users to combine the tools we provide for visual inspection as well as the image statistics, just in the manner in which the algorithm was developed in this work (S1 Appendix).

## 2. Generation of synthetic images

Synthetic images provide ground truth datasets to assess the systematic errors in the segmentation algorithm (Fig 3; [14]). We randomly allocated centroids of *N* objects in a three-dimensional continuous space, given by *L_x_* × *L_y_* × *L_z_* μm^3^. Let $X_{j}=\left(x_{j},y_{j},z_{j}\right)$be the centroid of an object *j*, (*j*=1, 2,…,*N*). To avoid severe overlap between two objects, we simulated the positions of objects using equation of motions with the Lennard-Jones potential [15]

$dX_{j}/dt=-\nabla U_{j}$,

where $U_{j}=\sum_{\begin{matrix} i=1, \\ i\neq j \end{matrix}}^{N} \varepsilon{}_{LJ}\left[\left(R/r_{ji}\right)^{3}-R/r_{ji}\right]$ and $r_{ji}=\left|X_{i}-X_{j}\right|$.

We set *ε_LJ_* = 0.1 and *R* = 8.5 μm, and simulated the equation of motion for 10 time units. We defined the density of objects as *ρ* = *N*/(*L_x_L_y_L_z_*), and varied *N* to obtain the representative densities close to real embryonic images. We approximated the shape of the nuclei as an ellipsoid with shape parameters, *a* = 4μm, *b* = 2.5μm and *c* = 2.5μm based on the measured average size of a typical nucleus from the embryonic data, which also determine its volume. We assigned the orientation of an object *j* using the Euler angle (*φ_j_*, *θ_j_*, *ψ_j_*) chosen from a uniform distribution, and locally rotated their axes as:

(S8)

For the synthetic images in Fig 3 in the main text, we used a uniform distribution between 0 and π/4 for *φ_j_* and *θ_j_*, and a distribution between 0 and π/2 for *ψ_j_*. For the synthetic image with random orientation in S3 Fig, we used a uniform distribution between 0 and 2π for *φ_j_*, *θ_j_* and *ψ_j_*. In the synthetic image with perfectly aligned objects in S3 Fig, we set *φ_j_* = *θ_j_* = *ψ_j_* = 0.

The signal intensity *I_j_*(*x,y,z*) at a given position (*x,y,z*)*,* emitted by an object *j*, was modeled as:

$I_{j}\left(x,y,z\right)=\frac{I_{0}}{2I_{n}}\left(\tanh\left[\sigma_{I}\left\{1-\left(\frac{x'}{a}\right)^{2}-\left(\frac{y'}{b}\right)^{2}-\left(\frac{z'}{c}\right)^{2}\right\}\right]+1\right),$ (S9)

where *I*_0_ is the maximum intensity at the centroid of the object *j*, *σ_Ι_* represents the steepness of an edge of the object and *I_n_* is the normalization constant, *I_n_* = (tanh[*σ_Ι_*]+1)/2. We set *σ_Ι_* = 2.0. Since there were *N* objects in the 3D space, the intensity value at a position (*x*, *y*, *z*) was given as a function of *I*_1_(*x*, *y*, *z*), *I*_2_(*x*, *y*, *z*), …, and *I_N_*(*x*, *y*, *z*). We defined the intensity value at the position (*x*, *y*, *z*) as:

$I\left(x,y,z\right)=I_{b}+\max\left[I_{1}\left(x,y,z\right),I_{2}\left(x,y,z\right),....I_{N}\left(x,y,z\right)\right],$ (S10)

where *I_b_* is a background intensity. To determine *I*_0_ and *I_b_*, we measured the average intensity at the center of nucleus *I_m_* and background intensity in the embryonic images. Then, we set *I_b_ =Ĩ_b_* and *I*_0_ *= I_m_ – Ĩ_b_*. In our 16-bit embryonic images, *I_m_* = 42089 and *I_b_ =*3474.

The image intensity profiles was discretized using the voxel spacing in each direction, Δ*x* =0.691μm, Δ*y* =0.691μm and Δ*z* =1.75μm, same as that of images obtained from the confocal microscope. The discrete spatial coordinate of the three-dimensional space can be written as where *k_ζ_* = 1, 2,…, *L_ζ_/Δζ* ,(*ζ* = *x*, *y* or *z*). We considered the intensity value at the center of the voxel (*k_x_*, *k_y_*, *k_z_*) as its intensity:

$I_{d}\left(k_{x},k_{y},k_{z}\right)=I\left(\frac{\Delta x}{2}\left(2k_{x}-1\right),\frac{\Delta y}{2}\left(2k_{y}-1\right),\frac{\Delta z}{2}\left(2k_{z}-1\right)\right).$ (S11)

We used the gamma distribution function to add noise in each voxel, given by, $f\left(x;\lambda,\omega\right)=x^{\lambda-1}\frac{e^{\frac{-x}{\omega}}}{\Gamma\left(\lambda\right)\omega^{\lambda}},$

where *λ* and *ω* are the shape and scale parameters, respectively and *Γ* is the gamma function. The mean of the gamma distribution (*λω*) was set to the value of intensity at each voxel, *λω = I_d_* (*k_x_, k_y_, k_z_*). We defined the signal-to-noise ratio (SNR) as the inverse of the coefficient of variation (CV) which is the ratio of the mean (*λω*) to the standard deviation (*σ_Γ_ = ωλ*^1/2^) of the gamma distribution, therefore SNR = *λ*^1/2^. The SNR represents the strength of intensity fluctuations within a nucleus. The shape and scale parameters in terms of SNR can be written as *λ* = SNR^2^ and *ω* = *I_d_* (*k_x_*, *k_y_*, *k_z_*)/SNR^2^, respectively. The intensity value for each voxel was generated from the gamma distribution using *λ* and *ω* to finally obtain 16-bit synthetic images. For complete source code, please see S2 Appendix.

## References

1. Lim JS, (1990) Two-Dimensional Signal and Image Processing. Englewood Cliffs, NJ, Prentice Hall, USA, 1^st^ edition.
2. Gonzalez RC, Woods RE, (1992) Digital Image Processing (Addison-Wesley Publishing Company, Inc. USA.1^st^ edition.
3. Shapiro LG, Stockman GC, (2001) Computer Vision, Prentice Hall, Englewood-Cliffs NJ, USA, 2001; 1^st^ edition.
4. Richardson WH, (1972) Bayesian-based iterative method of image restoration. J Opt Soc Am 62, 55.
5. Lucy LB, (1974) An iterative technique for the rectification of observed distributions. Astron J 79: 745–754.
6. Perona P, Malik J, (1990) Scale space and edge detection using anisotropic diffusion. IEEE Transactions on Pattern Analysis and Machine Intelligence. 12: 629–639.
7. Otsu N, (1979) A threshold selection method from gray-level histograms. IEEE Transactions on Systems, Man, and Cybernetics, 9: 62–66.
8. Haralick RM, Shapiro LG, (1992) Computer and Robot Vision, Addison-Wesley, Volume I, 28-48.
9. Hartigan JA, Wong MA, (1979) K-means clustering algorithm. Journal of the Royal Statistical Society, Series C, Applied Statistics 28, 100.
10. Amorim RC, Mirkin B, (2012) Minkowski metric, feature weighting and anomalous cluster initializing in k-means clustering. Pattern Recognition. 45: 1061-1075.
11. Press WH, Teukolsky SA, Vetterling WT, Flannery BP, (2007) Numerical Recipes: The Art of Scientific Computing, Section 16.1. Gaussian Mixture Models and k-Means Clustering. New York: Cambridge University Press, USA, 3^rd^ edition.
12. Figueiredo MAT, Jain AK, (2002) Unsupervised learning of finite mixture models. IEEE Transactions on Pattern Analysis and Machine Intelligence. 24: 381.
13. Akaike H (1980) Bayesian Statistics: Likelihood and the Bayes procedure, Valencia: University Press, 1^st^ edition.
14. Sbalzarini IF, Koumoutsakos P, (2005) Feature point tracking and trajectory analysis for video imaging in cell biology. Journal of Structural Biology. 151: 182-195.
15. Frenkel D, Smith B, (2002) Understanding Molecular Simulation. San Diego: Academic Press„ USA, 2^nd^ edition.
